# Validation of Heart Rate Extracted From Wrist-Based Photoplethysmography in the Perioperative Setting: Prospective Observational Study

**DOI:** 10.2196/27765

**Published:** 2021-11-04

**Authors:** Eveline Mestrom, Ruben Deneer, Alberto G Bonomi, Jenny Margarito, Jos Gelissen, Reinder Haakma, Hendrikus H M Korsten, Volkher Scharnhorst, R Arthur Bouwman

**Affiliations:** 1 Department of Anesthesiology Catharina Hospital Eindhoven Eindhoven Netherlands; 2 Clinical Laboratory Catharina Hospital Eindhoven Eindhoven Netherlands; 3 Department of Biomedical Engineering Eindhoven University of Technology Eindhoven Netherlands; 4 Expert Center Clinical Chemistry Eindhoven Eindhoven Netherlands; 5 Department of Personal Health Philips Research Eindhoven Netherlands; 6 Department of Electrical Engineering Eindhoven University of Technology Eindhoven Netherlands

**Keywords:** validation, heart rate, photoplethysmography, perioperative patients, unobtrusive sensing

## Abstract

**Background:**

Measurement of heart rate (HR) through an unobtrusive, wrist-worn optical HR monitor (OHRM) could enable earlier recognition of patient deterioration in low acuity settings and enable timely intervention.

**Objective:**

The goal of this study was to assess the agreement between the HR extracted from the OHRM and the gold standard 5-lead electrocardiogram (ECG) connected to a patient monitor during surgery and in the recovery period.

**Methods:**

In patients undergoing surgery requiring anesthesia, the HR reported by the patient monitor’s ECG module was recorded and stored simultaneously with the photopletysmography (PPG) from the OHRM attached to the patient’s wrist. The agreement between the HR reported by the patient’s monitor and the HR extracted from the OHRM’s PPG signal was assessed using Bland-Altman analysis during the surgical and recovery phase.

**Results:**

A total of 271.8 hours of data in 99 patients was recorded simultaneously by the OHRM and patient monitor. The median coverage was 86% (IQR 65%-95%) and did not differ significantly between surgery and recovery (Wilcoxon paired difference test *P*=.17). Agreement analysis showed the limits of agreement (LoA) of the difference between the OHRM and the ECG HR were within the range of 5 beats per minute (bpm). The mean bias was –0.14 bpm (LoA between –3.08 bpm and 2.79 bpm) and –0.19% (LoA between –5 bpm to 5 bpm) for the PPG- measured HR compared to the ECG-measured HR during surgery; during recovery, it was –0.11 bpm (LoA between –2.79 bpm and 2.59 bpm) and –0.15% (LoA between –3.92% and 3.64%).

**Conclusions:**

This study shows that an OHRM equipped with a PPG sensor can measure HR within the ECG reference standard of –5 bpm to 5 bpm or –10% to 10% in the perioperative setting when the PPG signal is of sufficient quality. This implies that an OHRM can be considered clinically acceptable for HR monitoring in low acuity hospitalized patients.

## Introduction

Timely recognition of deterioration in hospitalized patients is important because early intervention improves clinical outcomes of mortality and unplanned intensive care unit (ICU) admissions and reduces length of stay [1]. Especially in perioperative care, complications related to surgery limit effectiveness of the surgery and are associated with increased mortality and costs [2,3]. From previous studies, it is known that vital signs such as heart rate (HR) and respiratory rate are important indicators of critical illness and are often altered long before a deterioration is clinically apparent [4-6]. In general, patients’ vital signs are assessed multiple times a day in general wards. However, patients may deteriorate between the scheduled measurements [1]. Therefore, both remote and continuous monitoring of HR and respiratory rate is considered a promising tool for early detection of patient deterioration in the low acuity or home setting.

The gold standard for measurement of HR in the perioperative setting is the multiple-lead electrocardiogram (ECG). However, there are practical limitations to continuous measurements of vital signs using ECG due to the obtrusiveness and limited mobility of patients. Novel solutions to monitor vital signs have been proposed in the literature [7]. One of these novel solutions is the wrist-based optical heart rate monitor (OHRM). The OHRM has the advantage of offering unobtrusive, remote, and continuous monitoring. The photopletysmography (PPG) sensor in the OHRM has shown potential to provide robust peak detection from which HR may be calculated [8,9]. Validation studies have been presented on the accuracy of these devices in healthy participants [10-17]. However, it remains unclear whether these tools are also reliable for monitoring vital signs in patients during hospital stay. The robustness of an OHRM should be studied in hospitalized patients before it can be reliably adopted in a clinical setting. Few studies have been performed in hospitalized patients, and these included mainly stable ward patients [13,18,19]. To check the accuracy of the OHRM in the acute phases of disease, the study population should ideally experience some deterioration in HR during the study period. Hospitalized patients are a heterogeneous population where HR can be influenced by all kinds of pathologies, particularly during surgery, which induces hemodynamic, metabolic, endocrine, and immunological alterations [20,21]. The objective of this study was to assess the agreement between the HR extracted from a PPG sensor–based OHRM and that of the gold standard 5-lead ECG connected to the patient monitor during surgery and recovery.

## Methods

### Study Design

We used a prospective, nonrandomized, observational, single-center study design to examine the perioperative period. The study was performed in the Catharina Hospital in Eindhoven, the Netherlands, a tertiary hospital that performs an average of 20,000 surgical procedures annually. The study was reviewed and approved by the Medical Research Ethics Committees United (study #NL65134.100.18).

### Study Population

All adult patients scheduled for noncardiac surgery were screened by anesthesiologists for inclusion in the study. Patients were selected by the anesthesiologist on a weekly basis and informed of the study prior to the surgical procedure. In total, 203 patients were eligible for inclusion, and 100 patients signed informed consent. Cardiac surgeries were excluded since the required extracorporeal circulation and scheduled ICU admission would complicate analysis.

To obtain a representative case mix of patients undergoing surgery, patients were categorized and stratified based on the American Society of Anesthesiologists Physical Status Classification (ASA class) [22] and risk of the surgery [23]. Patients were divided into 2 groups: (1) low risk (ASA score I or II and low- or intermediate-risk surgery) and (2) high risk (ASA score III or IV and intermediate- or high-risk surgery). If the ASA score and risk were discordant (eg, ASA score IV and low-risk surgery), the ASA score took precedence over the surgical risk.

### Study Procedure

The measurements on the OHRM started as soon as the device was placed on the patient’s wrist in the holding area. The choice of wrist depended on the placement of the blood pressure cuff. Unless not otherwise possible, the OHRM was placed on the wrist of the arm opposite to the blood pressure cuff to prevent disturbance in the optical measurements of the cardiac pulse. The vital sign measurement started upon arrival in the operating room when sensor modules were connected to the patient monitor. Measurements continued during surgery (surgical phase). After completion of the surgery, the patient was disconnected from the patient monitor located in the operating room and transferred to the recovery room. Upon arrival in the recovery room, the patient monitor was reconnected to the patient monitor located in the recovery room, and measurements continued (recovery phase) until the patient was transferred to the general ward. Upon transfer, the patient monitor was disconnected, and the OHRM was removed from the patient’s wrist.

### Data Collection

**Figure 1:**
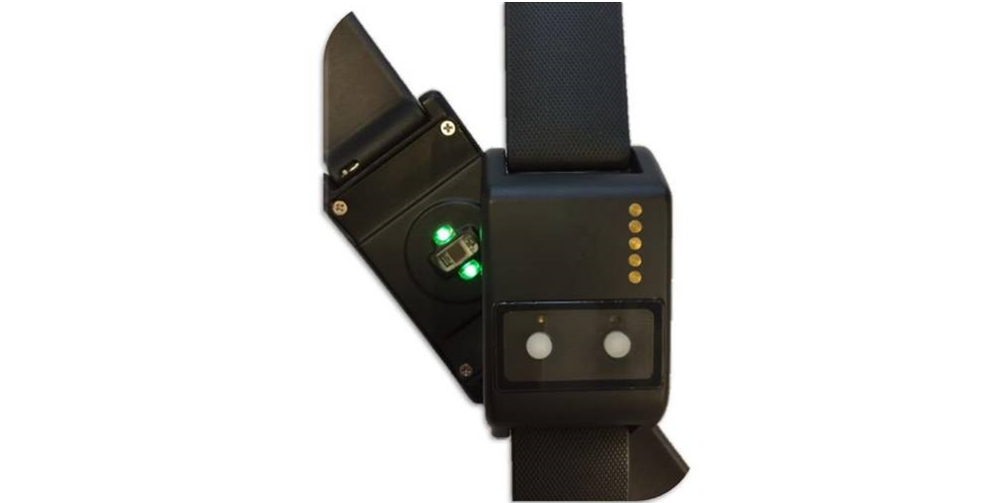
The wrist-worn optical heart rate monitor.

The wrist-worn OHRM was developed by Philips and equipped with a Philips Cardio and Motion Monitoring Module, which integrates a PPG and accelerometer sensor (Figure 1). PPG is an optical technique used to detect volumetric changes in blood in peripheral circulation. It continuously measures the reflectivity of the skin in the green part of the light spectrum in combination with the 3-axial acceleration of the body part where it is located. Accelerometry is a technique used to quantify movement patterns through the detection of rotational and translational acceleration. The sampling frequency of both the PPG and accelerometer sensors was 32 Hz [24]. The patient monitor in both the operating and recovery room was a Carescape B850 (GE Healthcare) connected to a 5-lead ECG, pulse oximeter, body temperature sensor, and oscillometric cuff for noninvasive blood pressure measurements or an arterial line for invasive blood pressure measurements. All patient monitors were linked to a patient data collection system which logged data for every patient. The application used for logging data was AnStat (CarePoint). AnStat logs trends and waveforms with a sampling frequency of 100 Hz and events like administration of drugs.

Data on patient demographics were extracted from the electronic medical records.

### Data Processing

The HR from the 5-lead ECG was derived by the Carescape B850 patient monitor software. The HR from the OHRM was extracted from the logged PPG signal using an algorithm that was previously validated in healthy volunteers in various conditions of rest and physical activity [25]. In brief, the algorithm processed the PPG and motion signal simultaneously to derive HR and a quality index (QI) for the HR measurements with a 1-second interval. Both HR and QI were assessed in real time. The algorithm provided an output every second, but the data were processed using a sliding window of 5 seconds. The HR measurements from the ECG and PPG were synchronized using a cross-correlation function and visual inspection of the resulting overlapped time series. The QI characterized the confidence in the provided metric estimated by the algorithm itself. It was represented on a 5-point scale (from 0-4), where 0 denoted “lowest confidence/output unavailable” and 4 denoted “highest confidence.” The QI was determined by proprietary methods and used to provide a monotonically increasing relation between availability and reliability. The QI of the HR was typically influenced by the signal-to-noise ratio of the PPG signal, the ability of the algorithm to cope with motion artifacts, and the periodicity of the detected pulse signal.

A PPG-based arrhythmia detection algorithm [26] was also used to identify periods in which the PPG signal was not in accordance with a normal sinus rhythm. In brief, the arrhythmia detection algorithm first identifies interpulse intervals (IPIs) in a 30-second interval from the PPG signal and then rejects the IPI in presence of motion during the IPI period. The final set of IPIs in the 30-second period are then processed by a Markov Model to define the probability of atrial fibrillation (AF). In our study, if >50% of the detected IPIs in the 30-second interval were rejected by the algorithm, the interval was labeled as arrhythmia. For measurement intervals during which events of arrhythmias were detected by this algorithm, the QI was set to 0. To summarize the PPG signal coverage, each HR measurement was assigned to 1 of 3 categories: (1) good quality (QI=4), (2) low quality (QI≤3), and (3) arrhythmia. Only HR data associated with QI=4 were used in the agreement analysis. Coverage was measured as the ratio between the measurements with good quality and the entire measurement duration for a patient. If patients had less than 5 minutes of coverage during surgery or recovery, the session was excluded from analysis. The hospital health records were screened to find potential causes for patients that were excluded since this would indicate that the OHRM was not usable for these patients.

Bland-Altman plots were made to visualize the agreement between ECG and PPG HR [27]. Limits of agreement (LoA) and CIs of the LoA were calculated by taking into account both within- and between-patient variability [28]. The modified Bland-Altman method that estimates the limits of agreement with repeated measurements where the true value varies, as described by Zou [29], was used. The CIs of the LoAs were constructed using the method of variance estimate recovery (MOVER). In short, a 1-way random-effects model was used to model the difference *d_ij_* of the *j*-th measurement for the *i*-th patient as follows:

d_ij_ = d + a_i_ + e_ij_

Where *d* is the unknown true difference between the ECG and PPG HR. The difference *d* is either the difference between the PPG and ECG HR (ie, d = HR_PPG_ – HR_ECG_) or the percentage difference calculated by *d* = d_%_ = 
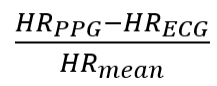
. *a_i_* and *e_ij_* are zero-mean normally distributed, with variance 
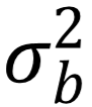
 and 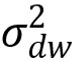 corresponding to the true between- and within-patient variances, respectively. The bias is estimated by 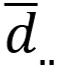, where 
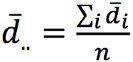
 and 
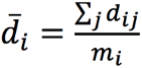
 and *m_i_* is the number of pairs per patient. The between- and within-patient variances were estimated by 
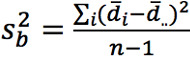
 and 
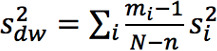
 where 
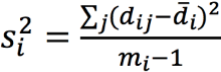
. 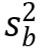 and 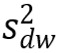 were summed to obtain an estimate of the total variance 
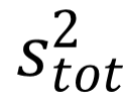
. The 95% LoA values were then calculated by 
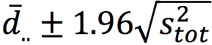
. CIs around the LoA values were estimated by the MOVER [29]. Bland-Altman analysis was conducted for both absolute difference and the percentage difference in HR between PPG and ECG. The HR evaluation was compared to the reference standard [30], which requires an accuracy of –5 bpm to 5 bpm or –10% to 10% (whichever is largest).

## Results

### Characteristics and Coverage

A total of 100 patients were included. One patient was excluded because the patient monitor data were missing due to technical difficulties. Recovery data of 1 patient were missing because this patient was transferred to the ICU immediately after surgery. Three patients had too few (<5 minutes) good quality PPG measurements during both the surgery and recovery phase and were therefore omitted from the agreement analysis. Another 12 patients had <5 minutes of good quality measurements during either the surgery or recovery phase, but only the respective phase was omitted from the agreement analysis. Patient demographics are shown in Table 1.

**Figure 2:**
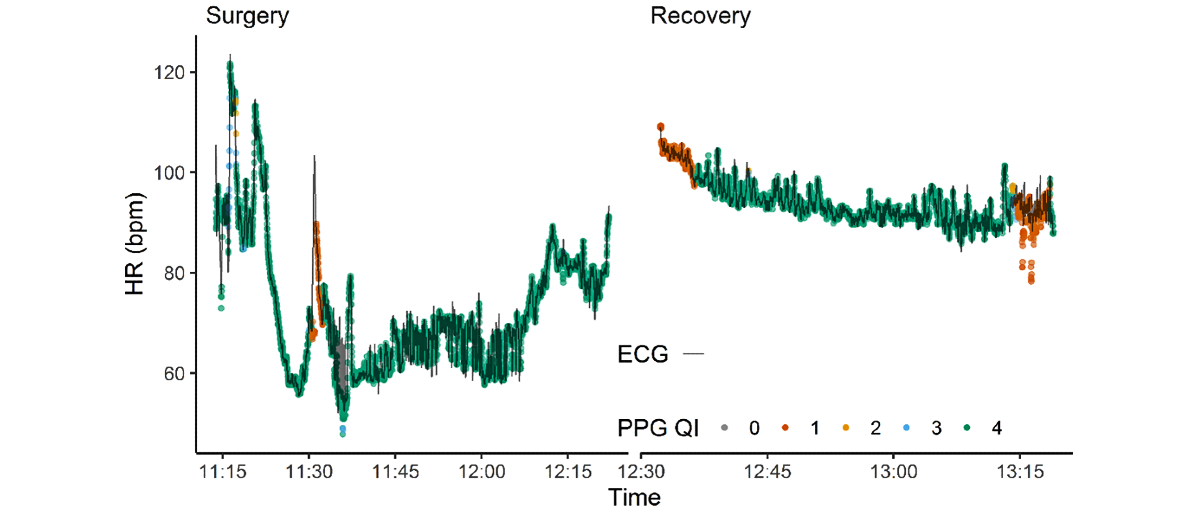
Example of data captured for a representative patient in the study. The ECG signal is represented by the gray line and the individual PPG measurements by the colored points. The QI of the PPG signal is represented by a different color which ranges from 0 (lowest quality) to 4 (highest quality). bpm: beats per minute; ECG: electrocardiogram; HR: heart rate; PPG: photopletysmography; QI: quality index.

**Figure 3:**
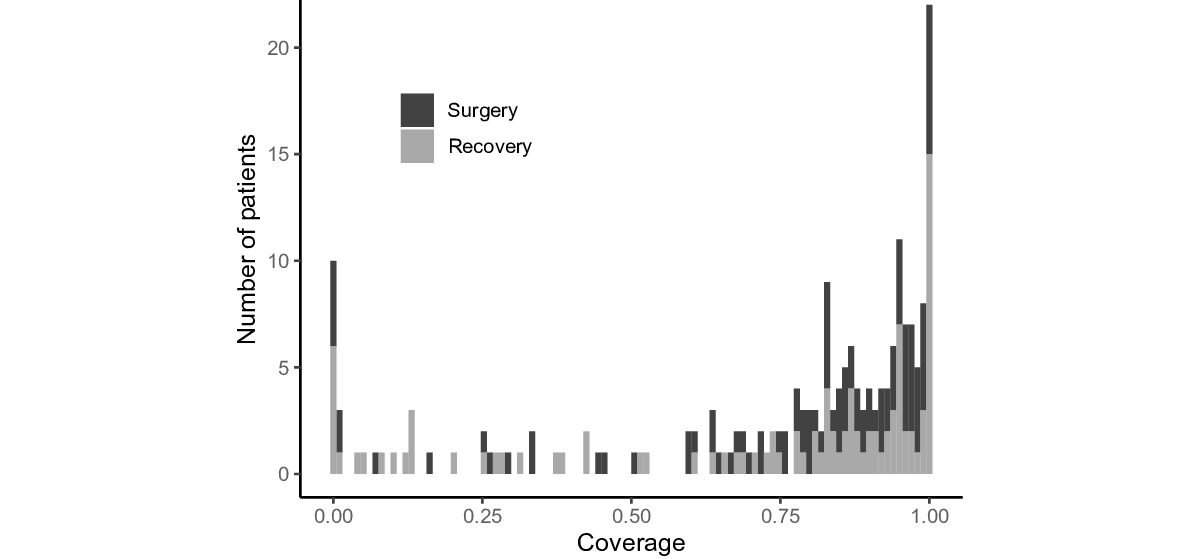
Histogram with distribution of coverage fraction (ie, proportion of recorded data that corresponds to a photopletysmography signal with good quality).

An example of the data that were captured for each patient in the study is shown in Figure 2. In total, 159.08 hours of data were captured during surgery, 76.5% (121.7/159.1 hours) of which were of good quality (QI = 4), and 112.59 hours of data were captured during recovery, 74.4% (83.8/112.6 hours) of which were of good quality. Coverage varied between patients (Figure 3). Median coverage was 86% (IQR 65% to 95%) and did not differ significantly between surgery and recovery (Wilcoxon paired difference test *P=*.17). Coverage statistics are shown in Table 2.

**Table 1:** Patient demographics.

Demographic variable		Value
Total participants, n		99
Age in years, median (IQR)		58.0 (44.5-68.0)
Male, n		36
BMI (kg/m2), median (IQR)		28.7 (24.8-37.1)
**ASA-PS^a^ score, n**		
	I	10
	II	39
	III	45
	IV	5
**Surgical risk, n**		
	High	9
	Intermediate	63
	Low	27
Diabetes, n		7
Hypertension, n		37
Hypercholesterolemia, n		21
Previous stroke or TIA^b^, n		13
Structural heart disease, n		8
Atrial fibrillation, n		8
**Wrist device location, n**		
	Left	45
	Right	53
	Unknown	1
**Surgery type, n**		
	Bariatric	22
	Gastroenterological	8
	Neurological	3
	Orthopedic	31
	Plastic	7
	Thyroid	1
	Urogenital	17
	Vascular	10
Surgery duration (min), median (IQR)		87.0 (48.0-115.0)
Recovery duration (min), median (IQR)		58.0 (41.2-78.0)

^a^ASA-PS: American Society of Anesthesiologists Physical Status.

^b^TIA: transient ischemic attack.

**Table 2:** Coverage statistics of total hours for analyses including all patients.

Characteristics of the collected data	Surgery	Recovery	Surgery and recovery
Total hours, n	159.6	112.2	271.8
Good quality PPG^a^ (hours), n (%)	124.1 (78.0)	83.8 (74.4)	207.9 (76.5)
Low quality PPG (hours), n (%)	33.3 (21.0)	28.7 (25.5)	62.0 (22.8)
Arrhythmia (hours), n (%)	1.7 (1.1)	0.2 (0.2)	1.9 (0.7)

^a^PPG: photopletysmography.

### Bland-Altman Analysis During Surgery

The mean bias was –0.15 (SD 0.05) bpm and –0.20% (SD 0.06) for the PPG-measured HR compared to the ECG-measured HR, where the LoA (including the SE) fell within the reference standard of –5 bpm to 5 bpm and –10% to 10% (Table 3).

**Table 3:** Bland-Altman analysis results during surgery.

Results	Difference in bpm^a^	Difference in percentage
Bias, mean (SE)	–0.15 (0.05)	–0.20 (0.06)
SD of differences	1.50	2.34
Lower LoA^b^ (95% CI)	–3.08 (–2.99 to –3.19)	–4.79 (–4.92 to –4.66)
Upper LoA (95% CI)	2.79 (2.69 to 2.89)	4.39 (4.26 to 4.53)
Within-patient variance	2.04	5.12
Between-patient variance	0.20	0.37
Intraclass correlation coefficient	0.09	0.07

^a^bpm: beats per minute.

^b^LoA: limits of agreement.

### Bland-Altman Analysis During Recovery

The mean bias was –0.10 bpm (SD 0.04) and –0.14% (SD 0.04) for the PPG-measured HR compared to the ECG-measured HR, where the limits of agreement (including the SE) fell within the reference standard of –5 bpm to 5 bpm and –10% to 10% (Table 4).

**Table 4:** Bland-Altman analysis results during recovery.

Results	Difference in bpm^a^	Difference in percentage
Bias, mean (SE)	–0.10 (0.04)	–0.14 (0.04)
SD of differences	1.38	1.93
Lower LoA^b^ (95% CI)	–2.80 (–2.72 to –2.87)	–3.92 (–3.83 to –4.01)
Upper LoA (95% CI)	2.59 (2.52 to 2.67)	3.64 (3.56 to 3.74)
Within-patient variance	1.78	3.56
Between-patient variance	0.11	0.16
Intraclass correlation coefficient	0.06	0.04

^a^bpm: beats per minute.

^b^LoA: limits of agreement.

## Discussion

A wrist-worn OHRM may be able to provide continuous unobtrusive HR monitoring in the low acuity care or home settings. To determine this, the validity of OHRM-derived HR must first be assessed in a representative target population and compared to the gold standard 5-lead ECG. In this study, the agreement between the HR derived from an OHRM and a 5-lead ECG connected to a patient monitor was assessed for a representative patient population during the perioperative period. The OHRM could provide an accurate HR (–5 bpm to 5 bpm and –10% to 10% compared to the ECG-derived HR) during both the surgical and recovery phase when the PPG signal was of good quality. A vast majority (121.7/159.1 hours, 76.5%) of the PPG signal was good quality.

Given the hemodynamic changes during the perioperative period and the diversity in surgical procedures, a technical validation, as performed in this study, is essential before the OHRM can be introduced into clinical practice. Very few studies were found in the literature that validated wrist-worn OHRMs in hospitalized patients. One study with a goal of early warning detection using an OHRM was performed in patients during and after discharge from the ICU [13]. The OHRM was a personal fitness tracker, and 24 hours of monitoring started in the ICU while patients were still being monitored by means of a continuous ECG. The authors concluded that personal fitness tracker–derived HRs were slightly lower than those derived from continuous ECG monitoring and not as accurate as pulse oximetry-derived HRs. A feasibility study was performed by the same research group regarding bradycardia and tachycardia detection in the same population [18]. The authors stressed in both studies the importance of subgroup analysis of patients not in sinus rhythm since this negatively impacted measurement accuracy. This corresponds to the findings in our study where measurements during arrhythmia were of low quality.

Another study was designed for AF detection, but also showed good results in sinus rhythm in patients undergoing elective cardioversion for AF [31]. There were fewer patients (N=20) included than in our study, and the agreement analysis was based on QRS intervals as the reference, with a mean difference of 1.3 ms being found between ECG and PPG. Other studies were performed in healthy participants and focused on assessing accuracy during physical activity [11,12,14,16,17,32-34]. However, the results obtained in these studies cannot be translated to our results since surgery was the underlying cause for changes in HR in our study and not physical activity. Factors influencing HR during surgery are hemodynamic changes induced by anesthesia, intraoperative factors such as blood loss and hypothermia, or involvement of vital organs in the area of surgery. Results of previous studies did conclude that motion artifacts remain a challenge in OHRMs. In this study, motion artifacts were less likely to occur since patients were mostly immobilized. Nevertheless, motion artifacts are relevant to consider if the OHRM is to be used in the future for remote monitoring of patients.

The agreement between the ECG- and PPG-derived HR was within the LoA of –5 bpm to 5 bpm and –10% to 10% (whichever was largest) both during surgery and recovery. However, this only applied when the quality of the PPG signal was labeled as “good.” Moreover, a vast majority (during surgery: 121.7/159.1 hours; during recovery: 76.5%; 83.8/112.6, 74.4%) of the PPG signal was good quality. Ideally, the coverage should be 100%, but this may not be realistic since a poor signal-to-noise ratio in the PPG measurements can perturb the detection of a sinus rhythm. Arrhythmias such as ectopic beats, AF, premature ventricular or atrial complex, and paced beats also contributed to a reduction of measurement coverage of the OHRM. This is confirmed by the fact that patients with a medical history of AF had lower overall coverage compared to patients without previous diagnosis of AF, resulting in 25% versus 85% overall coverage, respectively. This was also true for those patients with severe congenital heart disease where median coverage was 47% versus 85% for patients without structural heart disease. Finally, a very small group of patients had an extremely low coverage, but a consequently large influence on the mean coverage. Median coverage was higher, with 85% being good quality data. Exclusions of patients in this study should be taken into account as well when clinical applicability of the OHRM is assessed. Furthermore, 3 patients were excluded since <5 minutes of data were captured in total, which could be explained in 1 case by serious congenital heart disease which involved aberrant anatomy. Another 12 patients with <5 minutes of good quality data during surgery or recovery were also excluded. As the reference standard, ECG is considered capable of providing 100% coverage. However, in clinical practice, this is most likely not the case since ECG HR detection can also fail in the presence of the aforementioned abnormalities.

The limitations of this study are the following. Despite a heterogeneous group of elective procedures and hospital setting, no general ward patients were included. Nevertheless, translation of our findings to patients in the general ward is reasonable as patients are transitioning from immobile to a more moveable state during stay in the recovery room. By using a 1-way random-effects model, the between- and within-patient variance was quantified to explore the effect of heterogeneity of the study group. As indicated by Hamilton and Lewis [35], not accounting for repeated measures can lead to a falsely narrow LoA, mainly with a small number of patients and a large number of measurements per patient. Both the mean bias and between-patient variance are weighted according to the number of observations available for each patient. Hence, patients with more observations will contribute more to the final results. As the distribution of observation times was not normal, some patients contributed substantially more than did others, and results could have been biased to these patients. It is also worth mentioning the assumptions underlying the 1-way random-effects model. Specifically, the model assumes that repeated differences on a single patient are independent and that the within-patient variance of these differences is constant and the same for all patients. First, the independence assumption could have been too strong since hemodynamic changes occurred during surgery or recovery which could have led to autocorrelation in the HR and subsequent differences arising between the PPG- and ECG-derived HR. The effect of autocorrelation on the within-patient variance is unknown, and further studies are needed to take autocorrelation into account [28]. Second, the assumption of homoscedasticity was not formally tested, and it could have been the case that the variance of the differences increased with higher HR. Finally, the possible influence of surgery-specific factors, such as electrosurgical instruments causing interference was not investigated.

With this study in hospitalized patients, we gained knowledge on the influence of the oscillometric blood pressure cuff which disturbs the measurements by compromising the blood flow. Although both nurses and patients experience the wireless and unobtrusive wristband as an advantage, the OHRM will still have to find a way into local workflow. Before early warning systems can be incorporated into timely detect deterioration, clinical studies to define the predictive value of continuous HR monitoring for deterioration in hospitalized patients other than the ICU or operating room are first needed. Although our own and previous studies might not have found equal accuracy compared to the gold standard, there is still more opportunity to produce benefit during the acute phases of illness, which otherwise may go unnoticed in the general ward with monitoring of the HR 2 to 3 times daily. Although the use of an ORHM for deterioration detection seems less time-consuming for nurses, it still remains uncertain what the false alarm rate will be or how much time practical procedures, such as charging the battery of the OHRM, will take. From a practical point of view, placement of the wristband is made problematic by intravenous or arterial lines, identification bands, or bandages on the wrists.

In summary, the current study found that the OHRM is clinically acceptable when good quality data are captured and in settings when high-intensity monitoring, such as in the ICU or operating room, is not mandatory. The OHRM seems less suitable for patients with congenital anatomical changes of the heart or patients with arrhythmias. When the OHRM captures a significant amount of low-quality data in a patient, the suggestion would be to use another monitoring type to ensure safe monitoring. Since the OHRM can report the quality of the PPG signal instantaneously, the decision to switch to ECG monitoring can be made immediately. The reliability of an OHRM to measure HR in patients known to suffer from arrhythmias or structural heart disease requires further research.

In conclusion, this study shows that an OHRM equipped with a PPG sensor can measure HR within the ECG reference standard of –5 bpm to 5 bpm and –10% to 10% in the perioperative setting when the PPG signal is of sufficient quality. This implies that an OHRM can be considered clinically acceptable for HR monitoring in low acuity hospitalized patients and may provide the basis for future studies for remote, unobtrusive, continuous monitoring for timely recognition of deterioration.

## Abbreviations

AF: atrial fibrillation
ASA-PS: American Society of Anesthesiologists Physical Status
bpm: beats per minute
ECG: electrocardiography
HR: heart rate
ICU: intensive care unit
IPI: interpulse interval
LoA: limits of agreement
MOVER: method of variance estimate recovery
OHRM: optical heart rate monitor
PPG: photoplethysmography
QI: quality index
